# Effect of different fat and protein levels in calf ration on performance of Sahiwal calves

**DOI:** 10.5713/ajas.18.0604

**Published:** 2019-02-07

**Authors:** Bharti Sharma, Prapti Nimje, S. K. Tomar, Dipak Dey, Santu Mondal, S. S. Kundu

**Affiliations:** 1 Indian Council of Agricultural Research (ICAR), National Dairy Research Institute (NDRI), Animal Nutrition Division, Karnal -132001 (Haryana), India; 2 Indian Council of Agricultural Research (ICAR), National Dairy Research Institute (NDRI), Livestock Production and Management, Karnal -132001 (Haryana), India

**Keywords:** Calf Starter, Growth, Nutrient Intake, Immunity Levels, Sahiwal Calves

## Abstract

**Objective:**

The current study was carried out to examine the response of different levels of fat and protein in calf starter on nutrient utilisation, nitrogen metabolism, weight gain, blood parameters, and immunity level in pre-ruminant calves.

**Methods:**

Twenty four calves (5 days old) were divided into six groups in a 2×3 factorial design, with two levels of fat (10% and 14%) and three levels of protein (18%, 21%, and 24%). The calves were kept in individual pens for 120 days and fed with whole milk (1/10th of body weight) and calf starter ad-libitum. Daily dry matter intake was recorded; whereas body weight was taken on fortnightly basis to calculate average daily gain. During the growth trial blood samples were collected at 30 days interval to estimate blood glucose, albumin, total protein, total leucocyte count, total immunoglobulins and immunoglobulin G levels. A metabolic trial of seven days was carried out to find out the digestibility of different nutrients.

**Results:**

The dry matter intake was reduced (p<0.05) with higher fat and protein levels whereas feed conversion efficiency was improved (p<0.05) with higher protein level. Different levels of fat and protein in calf ration did not affect average daily gain in calves. The dry matter, organic matter, and crude protein digestibility were significantly (p<0.01) higher with increased level of protein. The nitrogen retention was also significantly higher (p<0.05) at 24% protein level, similarly the total immunoglobulin was significantly (p<0.05) high in higher protein fed groups, showed better immunity.

**Conclusion:**

The present finding suggested that 10% fat and 18% protein level of calf starter could be used in Sahiwal calves for optimum performance in terms of weight gain and immunity.

## INTRODUCTION

Health and performance of claves is the main focus area in calf rearing programme at any dairy herd to maintain future stock. Applying a suitable feeding strategy can maximize daily weight gain and better health of calves. Weaning of calves on milk feeding reduces the concentrate feeding, slower rumen development which ultimately decreases the feed intake and growth in post weaning stage [1]. In the modern dairy industry feeding of early-weaned calves with milk replacer is an effective way to raise replacement cattle [2]. Consumption of dry feed in young calves is a priority to stimulate the reticulorumen development [3]. Dry feed consumption by calves leads to reduction in milk feeding period; hence decrease the cost of raising replacement stock. Pre-ruminants depend solely on dietary sources of protein until the functional rumen is developed. Use of milk or animal proteins in milk replacer or calf starter is costly; hence plant protein sources can be used in place of milk or animal protein sources. The plant protein and fat sources like soybean meal, ground corn, oats meal, full fat soybean etc. can be utilized as ingredients in calf concentrate mixture [4]. The calf starter should have 20% crude protein (CP) (dry matter [DM] basis; 18% as fed basis) as per [5]. A report said that calves fed with 22% CP in starter feed were more efficient for BW gain than calves fed 18% CP in starter [6]. Another study found that protein levels of starter feed did not significantly affect feed intake and performance of calves [7]. Lohakare et al [8] reported that live weight gain and feed intake of calves was not affected when calves fed with different levels of proteins (19.46, 16.22, and 24.815) in diets. Fat supplementation provides energy for growth of calves. Feeding of starter feed containing more than 7.3% fat DM or 20% has been reported to decrease DM intake and BW gain [9,10]. Increasing the fat content (11.2% DM basis) of starter feed leads to decrease in feed intake but improvement of growth rate in Holstein calves [11]. Under Indian conditions farmers raise calves on milk which increase the cost of production, hence calf starter made with low cost protein and fat sources may reduce the cost of production.

Scientific literature regarding the effects of fat and protein contents in calf starter on DM intake, growth performance, and health parameters in Sahiwal calves is limited while the breed is very popular among farmers in India. This study was conducted to compare the effects of feeding two different levels of fat with three different protein levels on feed consumption, BW gain, health and selected blood metabolites in Sahiwal calves.

## MATERIALS AND METHODS

### Selection, rations and management of calves

Twenty four Sahiwal calves (5 days old) were selected and a feeding trial was conducted at Livestock research centre, ICAR-NDRI, Karnal, India. The calves were divided into six groups (four calves in each group) having similar average BW (21.00 kg). Six different calf starters were prepared with two levels of fat (F_1_: 10% and F_2_: 14%) having three levels of protein (P_1_: 18%, P_2_: 21%, and P_3_: 24%). Calves were fed *ad libitum* with either of the six different calf starter i.e. F_1_P_1_, F_1_P_2_, F_1_P_3_, F_2_P_1_, F_2_P_2_, and F_2_P_3_. Calf starters were totally formulated from locally available vegetative sources and their ingredients proportion is presented in Table 1. Calves were housed in individual pens on concrete floor housing. Calves were fed with Colostrum (10% of BW) upto 5 day of age, and then from 6 to 90 days of age whole milk feeding (10% of BW) was done. Calf starter and green fodder were offered to calves *ad libitum* from 15th day of age and feeding schedule of calves is presented in Table 2.

The feeding trial was conducted for 120 days and calves fed twice a day in morning and evening at 9:00 am and 4:00 pm, respectively. The fresh and clean water was made available round the clock. Normal deworming and vaccination schedule was followed to maintain the health of calves as per institutional schedule. At the end of feeding trial, a seven days metabolic trial was carried out to work out nutrient digestibility and N balance.

### Sample collection and chemical analysis

The DM intake was recorded daily for individual animal. The samples of feed offered and residue left were collected and pooled separately for individual animal. For biochemical parameters like glucose, total protein, albumin and globulin as well as total immunoglobulins and immunoglobulin G (IgG), blood sample collected from jugular vein and plasma separated by centrifuge machine at 3,000 rpm for 10 min. The plasma samples were stored at −20°C until the analysis.

Digestibility of nutrients was estimated by difference method. During metabolic trial the samples of feed offered, residue left, dung and urine were collected and analyzed as per procedure [12] for organic matter (OM), CP, and ether extract (EE). Neutral detergent fiber (NDF) was assayed without α-amylase [13].

### Estimation of crude protein and total digestible nutrient requirement of Sahiwal calves

During the feeding trial period BW, average daily gain (ADG) and nutrient intake of Sahiwal calves were recorded and data were analysed at fortnightly interval. The fortnightly CP intake (g/kg^0.75^) and total digestible nutrient (TDN) intake (g/kg^0.75^) were regressed linearly from ADG (g/kg^0.75^) for the determination of CP and TDN requirements for maintenance and growth. Regression equations were developed and CP and TDN requirements for maintenance were obtained by putting the ADG value at zero.

### Statistical analysis

The experiment was designed as a 2×3 factorial and data were analysed using the general linear model procedure of the Statistical Analysis System Institute [14]. To test the effects of protein and fat concentration, fixed effects in the model were protein concentration, fat concentration, and their interaction. The significant differences were considered at α≤0.05 and no significant at α>0.05.

The model of this experiment is as follows:

$${\text{Y}}_{\text{ijk}}=\mu +{\text{P}}_{\text{i}}+{\text{F}}_{\text{j}}+{\text{PF}}_{\text{ij}}+{\text{E}}_{\text{ijk}}$$

Whereas Y_ijk_ was the feeding effect on calves, μ was the mean value, P_i_ was an effect of protein, F_j_ was the effect of fat, PF_ij_ was the interaction of protein and fat and E_ijk_ is error value.

## RESULTS

### Blood plasma biochemical parameters in Sahiwal calves

The summary of blood plasma parameters during the growth trial is presented in Table 3. Albumin and IgG levels did not differ significantly among the groups. Blood plasma parameters did not vary significantly with change in fat levels of calf starter except total immunoglobulin levels. The glucose and total protein levels were increased significantly with higher levels of protein (24%) than other levels of protein (18% and 21%), whereas total leucocyte count was significantly (p<0.01) reduced with increased levels of the protein. Total immunoglobulin levels were increased significantly with increase in fat and protein levels of ration that means immunity of calves improved with feeding of higher levels of fat and protein.

### Nutrient digestibility and nitrogen balance in Sahiwal calves

The different nutrient digestibility coefficient among the groups is presented in Table 4. There was a significant change in nutrient digestibility coefficient among the groups at different levels of protein but not in different levels of fat. The digestibility of DM, OM, and CP were significantly reduced at 18% protein level than 21% and 24% protein level; whereas the digestibility of EE was significantly improved at 18% protein level. The digestibility coefficient of NDF and acid detergent fiber (ADF) did not differ significantly among groups.

The intake, outgo, absorption and retention of nitrogen (N) in Sahiwal calves are presented in Table 5. There was no significant effect of fat and protein content of ration on N intake, N voided in faeces and urine, total N outgo and N absorption. The N retention in calves was significantly increased with increased protein level (p<0.05). The absorbed N and retained N as the percentage of N intake were also high with higher levels of protein.

### Nutrient intake and weight gain in Sahiwal calves

The data on DM, CP, metabolisable energy (ME), TDN intake as well as ADG and feed conversion efficiency (FCE) during growth trial is provided in Table 6. Fat and protein levels of calf starter affected the DM intake in calves significantly. The DM intake was higher in group fed with calf starter having 10% fat and 18% protein level. The CP, ME, and TDN intake did not differ significantly among the groups. There was no significant effect of different levels of protein and fat on ADG. The FCE was significantly higher at 24% level of protein but fat level did not have significant impact on it.

### Requirement of crude protein for maintenance and growth of Sahiwal calves

**Figure 1:**
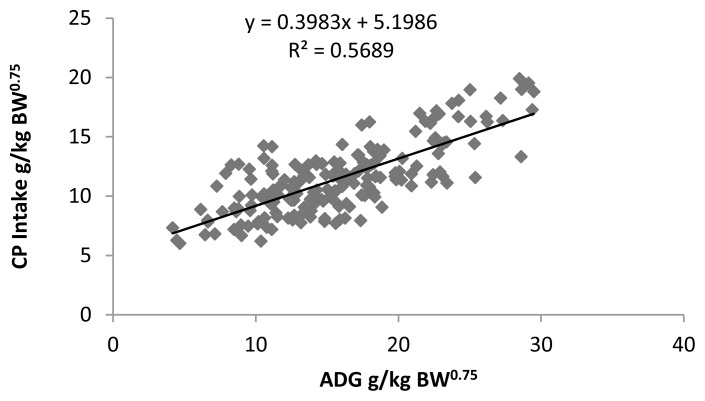
Relationship of CP intake (g/kg BW^0.75^) with ADG (g/kg BW^0.75^) of Sahiwal calves. CP, crude protein; BW, body weight; ADG, average daily gain.

To find out the CP requirement of Sahiwal calves for maintenance and growth the CP intake (g/kg^0.75^) was regressed linearly with ADG (g/kg^0.75^). The regression equation is presented below (Figure 1).

$$\text{y}=0.3983\text{x}+5.1986\;({\text{R}}^{2}=0.5869,\text{p}<0.01)$$

Where, y = CP intake (g/kg^0.75^) of Sahiwal calves and x = ADG (g/kg^0.75^).

From the developed equation, the requirement of CP for maintenance and growth is 5.20 g/kg^0.75^ and 0.40 g/g ADG/kg BW^0.75^/d, respectively.

### Requirement of total digestible nutrient for maintenance and growth of Sahiwal calves

**Figure 2:**
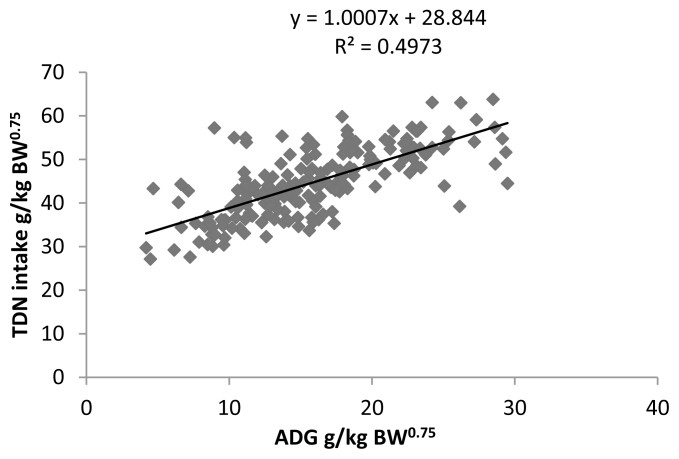
Relationship of TDN intake (g/kg BW^0.75^) with ADG (g/kg BW^0.75^) of Sahiwal calves. TDN, total digestible nutrient; BW, body weight; ADG, average daily gain.

The TDN intake (g/kg^0.75^) was regressed linearly from ADG (g/kg^0.75^) (Figure 2). The regression equation developed is as follows:

$$\text{y}=1.0007\text{x}+28.844\;({\text{R}}^{2}=0.4973,\text{p}<0.01)$$

Where, y = TDN intake (g/kg^0.75^) of Sahiwal calves and x = ADG (g/kg^0.75^).

From the structured equation, the TDN requirement of the growing calves for maintenance and growth is 28.8 g/kg^0.75^ and 1.0 g/g ADG/ kg BW^0.75^/d.

## DISCUSSION

### Blood plasma biochemical parameters in Sahiwal calves

The plasma albumin and IgG concentration did not vary with different levels of fat and protein in the present feeding trial. Similar to the present finding Daneshvar et al [15] reported no change in blood albumin levels after feeding calf starter containing 20% and 24% CP in the Holstein calves. There was no significant change observed in albumin levels after feeding the calves with different CP (23%, 25%, and 27%) contained milk replacer [16]. In the present study, glucose concentration increased with higher protein levels, which agrees with previous findings [17–19]. In contrary, Blome et al [20] found that glucose concentration was not changed significantly due to CP content in milk replacer. It was found that total protein levels increased following increased CP content in calf starter. Protein levels, when increased in calf starter, might have spared blood proteins from catabolism, thus temporarily increased blood protein levels. Similarly, glucose would also be spared and ultimately leads to increased immunoglobulin levels. Li et al [21] also found higher level of plasma total protein in claves fed with higher protein levels in milk replacer.

### Nutrient digestibility and nitrogen balance in Sahiwal calves

The digestibility of DM, OM, and CP in the present study improved with the increase in protein levels. This improvement in digestibility of DM, OM, and CP could be due to increase in quantity of better quality nutrients with increase in protein levels of calf starter. Chapman et al [22] reported that calves fed with milk replacer (26% CP, 18% fat) had greater nutrient digestibility for DM, OM and tended to be greater for CP digestibility than the control groups (20% CP, 20% fat). In contrary, feeding of calves with MR (27% CP and 17% fat) at pre-weaning stage leads to decreased OM digestibility of 11% at post-weaning stage [23]. The EE digestibility was higher in calves fed with lower protein level of ration in this experiment. The EE digestibility did not differ significantly between three groups of dairy calves fed with rations having low (18%), medium (22%) and high (26%) protein levels [21]. In the present study, NDF and ADF digestibility remain unaltered at different fat and protein levels of calf starter have similarity with earlier report of Mehra et al [24] who stated that NDF digestibility variation was non-significant as levels of energy and protein increased in experimental diets. The NDF and ADF did not differ significantly by dietary CP levels [25,26].

Nitrogen intake (NI), N voided through faeces, urine and total N outgo remained unchanged during experimental trial. Chapman et al [22] observed no differences in N intake in Holstein dairy calves fed with high protein milk replacer. The N absorption did not alter with various levels of fat and protein. In the present trial the N retained, N retained as percentage NI, retained N as percentage NI and retained N as the percentage of absorbed were significantly higher with increased protein level in calf ration. The similar kind of results was concluded by Blome et al [20].

### Nutrient intake and weight gain in Sahiwal calves

In the present study calves were fed milk at the rate of 10% of their BW but experimental rations feds *ad libitum*. The DM intake was reduced with higher fat and protein levels of calf starter. The decrease in DM intake with increase of CP and fat levels in rations could be due to increase in energy density of calf starter which satisfied the energy requirements of calves. Previous researcher Quigley et al [19] compared the effect of fixed amount feeding of a milk replacer (20% CP and 20% fat) with variable amount of a milk replacer (28% CP and 17% fat) feeding on calf starter intake, suggested that additional feeding of milk replacer decrease calf starter intake of calves up to 32 days age. In another study, lesser starter feed intake was observed when calves were supplemented with fat through milk replacer [27]. Similarly, Hill et al [28] found that starter intake by calves was decreased as the fat percentage of milk replacer increased. Lopes et al [29] recorded no variation in DM intake of calves when the calves received supplement having different levels of protein (8%, 19%, 30%, and 41% of CP).

The CP and ME intakes did not vary significantly with different levels of fat and protein in the present experiment. Similar to this study, Hill et al [16] concluded that ME intake among groups did not differ when the different groups of calves were fed with milk replacer containing 17% fat with different levels of CP (23%, 25%, 27%, and 29%) in a 56 days trial. In another report, 27% CP containing milk replacers with 4 different concentrations of fat (14%, 17%, 20%, and 23%) were fed and CP intake did not change significantly [28].

In the present study, increasing CP and fat content of starter feed (on DM basis) up to 24% and 14% respectively did not have a positive impact on performance of dairy calves. The genetic potential in Sahiwal might not be high enough to support increased gain due to high nutrient supply. Akayezu et al [30] stated that gain in BW was similar in calves fed with starter feed containing 19% and 22% CP from 4 to 56 days of age but these gain in BW were more than groups fed with 15% and 17% CP starter feed. Previous studies [31,32] reported that increasing the protein content above 20% of starter DM did not improve the performance of calves. Similarly, other studies concluded that performance of calves remained same at different CP levels of the starter [15,33]. Similar to present finding Araujo et al [11] found that fat levels of starter feed did not influence the ADG in Holstein calves. The FCE was increased with higher protein levels of diet represents better utilisation of calf rations in the present experiments. The FCE of present study was comparable with earlier reports. Blome et al [20] and Hill et al [16] reported that efficiency of gain increased linearly as dietary CP increased in milk replacers. Brown et al [34] also noted higher gain: feed ratio in calves fed with high protein diets.

## CONCLUSION

The present study revealed that increasing the protein and fat content of calf starter had no effects on calf performance and health. It can be concluded that 10% fat and 18% protein levels in starter feed on DM basis is sufficient for optimum growth performance of Sahiwal calves. Further studies are required to observe the effect of increasing CP and fat levels in calf starter on rumen development in Sahiwal calves.

## Figures and Tables

**Table 1:** Ingredient (%) composition of the calf rations used during trial

Ingredients (%)	10%1)			14%1)		
	
	18%2)	21%2)	24%2)	18%2)	21%2)	24%2)
Maize grain	35	24	30	35	32	30
DORB	10.1	18	8.7	10.1	7.3	2.5
Wheat bran	20	15	10	20	8.3	7.2
GNC	12	9	20	12	7.5	16
Full fat soya	13	25	23.3	13	32.4	32.5
Molasses	5	5	5	5	5	5
Prilled fat	1.9	1	0	1.9	4.5	3.8
Mineral mixture	2	2	2	2	2	2
Salt	1	1	1	1	1	1

DORB, deoiled rice bran; GNC, groundnut cake.

1) Fat levels.

2) Protein levels.

**Table 2:** Feeding schedule of calves

Age group	Whole milk	Calf ration	Green fodder offered
0–5 days	1/10th of body wt. (Colostrum)	Nil	Nil
6–30 days	1/10th body wt. (Max. 3 L)	Introduced gradually after 15 d of age	Offered (negligible consumption)
1–2 month	1/10th body wt. (Max. 3 L)	200–300 g	*Ad libitum*
2–3 month	Max. 3 L	300–400 g	*Ad libitum*
3–4 month	Nil	400–600 g	*Ad libitum*

**Table 3:** Effect of different levels of fat and protein on blood plasma parameters in Sahiwal calves

Parameters	10%1)			14%1)			Effect		
		
	18%2)	21%2)	24%2)	18%2)	21%2)	24%2)	Fat (F)	Protein (P)	F×P
Albumin (g/L)	19.95±2.57	13.30±1.29	14.01±4.00	11.41±0.64	15.23±1.71	11.75±1.34	NS	NS	0.0404
Immunoglobulin G (mg/100 mL)	2,536.54±862.28	1,821.89±70.52	1,812.50±12.53	1,833.23±61.46	2,525.23±905.16	1,902.38±196.44	NS	NS	NS
Glucose (mg/dL)	90.33^xy^±1.91	85.51^x^±4.35	90.89^y^±1.78	86.47^xy^±0.06	84.28^x^±5.37	94.04^y^±3.12	NS	0.0462	NS
Total protein (g/L)	48.80^x^±0.61	51.13^xy^±0.07	51.22^y^±0.69	51.07^x^±2.42	50.66^xy^±0.90	53.98^y^±0.31	NS	0.0390	NS
Total leucocyte count (10^3^/μL)	12.91^y^±0.44	12.47^y^±0.04	10.07^x^±0.13	11.26^y^±0.36	11.13^y^±0.30	11.46^x^±0.44	NS	0.0004	0.0002
Total immunoglobulins (mg/mL)	21.51^B^^xy^±0.65	22.08^B^^y^±0.44	22.19^B^^x^±0.41	23.37^A^^xy^±0.74	24.47^A^^y^±0.74	21.55^A^^x^±0.46	0.0100	0.0391	0.0191

NS, non significant.

1) Fat levels.

2) Protein levels.

A,B Capital letters in the same row, differ at p<0.05 by least square means for fat levels effect.

x,y Letters in the same row, differ at p<0.05 by least square means for protein levels effect.

**Table 4:** Effect of different levels of fat and protein on nutrient digestibility (%) in Sahiwal calves

Parameters	10%1)			14%1)			Effect		
		
	18%2)	21%2)	24%2)	18%2)	21%2)	24%2)	Fat (F)	Protein (P)	F×P
Dry matter	57.78^x^±2.07	60.43^y^±3.02	70.92^y^±4.59	62.16^x^±1.22	64.83^y^±0.71	61.90^y^±2.57	NS	0.0054	NS
Organic matter	59.21^x^±1.92	61.32^y^±3.01	72.16^y^±4.46	63.03^x^±1.43	68.29^y^±2.03	62.54^y^±2.52	NS	0.0023	NS
Crude protein	47.93^x^±6.14	45.55^y^±1.58	63.90^y^±4.25	43.77^x^±2.75	51.78^y^±3.80	47.49^y^±2.53	NS	0.0034	0.0467
Ether extract	86.49^x^±1.79	80.79^x^±3.99	80.17^y^±2.97	77.76^x^±4.40	60.58^x^±4.97	54.34^y^±5.63	NS	0.0000	NS
Neutral detergent fiber	49.72±3.22	52.84±2.42	63.93±7.10	54.45±2.45	54.60±1.48	57.18±5.42	NS	NS	NS
Acid detergent fiber	42.46±1.72	47.77±5.07	58.72±6.75	49.47±3.04	51.83±0.49	44.90±4.86	NS	NS	NS
Non-fiber carbohydrate	80.34^y^±3.44	70.16^x^±2.34	89.42^x^±1.20	90.13^y^±1.49	89.09^x^±3.97	91.23^x^±4.32	NS	0.0001	NS

NS, nonsignificant.

1) Fat levels.

2) Protein levels.

x,y Letters in the same row, differ at p<0.05 by least square means for protein levels effect.

**Table 5:** Effect of different levels of fat and protein on nitrogen balance in Sahiwal calves

Parameters	10%1)			14%1)			Effect		
		
	18%2)	21%2)	24%2)	18%2)	21%2)	24%2)	Fat (F)	Protein (P)	F×P
N intake (g/d)	28.06±2.21	37.36±8.13	42.23±6.66	30.29±1.64	29.67±0.12	34.26±3.56	NS	NS	0.0396
N voided in faeces (g/d)	15.24±0.78	18.82±2.43	15.37±3.29	14.66±1.81	16.69±0.88	17.87±1.05	NS	NS	NS
N voided in urine (g/d)	2.98±0.55	4.22±0.69	6.55±1.55	3.93±0.82	4.88±0.01	6.43±1.71	NS	NS	NS
Total N outgo (g/d)	18.22±1.31	23.03±3.10	21.92±2.75	18.59±2.61	21.57±0.87	24.30±2.57	NS	NS	NS
Absorbed nitrogen (g/d)	12.83±1.45	18.55±5.76	26.87±4.00	15.64±0.93	12.98±0.77	16.39±2.53	NS	NS	0.0127
N retained (g/d)	9.85^xy^±0.91	14.33^x^±5.07	20.31^y^±3.91	11.71^xy^±1.36	8.10^x^±0.76	9.96^y^±1.46	NS	0.0303	0.0320
Absorbed N as % of N intake	45.55^y^±1.58	47.93^x^±6.14	63.89^y^±4.25	51.78^y^±3.80	43.78^x^±2.75	47.49^y^±2.53	NS	0.0034	0.0467
Retained N as % of N intake	35.03^y^±0.49	36.42^x^±6.88	47.77^z^±1.58	38.95^y^±5.61	27.32^x^±2.67	29.05^z^±2.63	NS	0.0020	NS

NS, nonsignificant.

1) Fat levels.

2) Protein levels.

x,y Letters in the same row, differ at p<0.05 by least square means for protein levels effect.

**Table 6:** Effect of different levels of fat and protein on nutrient intake, average daily gain and feed conversion efficiency in Sahiwal calves

Parameters	10%1)			14%1)			Effect		
		
	18%2)	21%2)	24%2)	18%2)	21%2)	24%2)	Fat (F)	Protein (P)	F×P
DMI (g/100 kg BW)	2,405.49^A^^y^±230.46	1,954.93^A^^x^±182.7	1,910.79^A^^x^±135.75	1,901.73^B^^y^±168.32	1,955.33^B^^x^±160.01	1,751.13^B^^x^±215.65	0.0323	0.0257	NS
CPI (g/100 kg BW)	526.87±42.18	445.39±17.27	452.49±12.74	447.59±42.05	454.06±25.33	412.72±41.10	NS	NS	NS
ME intake (MJ/100 kg BW)	34.89±2.32	30.33±1.26	29.49±0.48	29.95±2.04	30.67±1.71	27.74±2.53	NS	NS	NS
TDN intake (g/100 kg BW)	2,031.21±287.86	1,715.75±81.41	1,607.56±45.21	1,683.05±116.18	1,721.39±96.21	1,588.24±95.08	NS	NS	NS
ADG (g/100 kg BW)	168.89±16.07	204.20±46.43	243.65±9.89	236.53±19.97	247.26±48.58	281.98±82.32	NS	NS	NS
FCE	0.26^x^±0.02	0.32^y^±0.04	0.41^y^±0.01	0.40^x^±0.05	0.42^y^±0.04	0.44^y^±0.11	NS	0.0151	NS

NS, nonsignificant; BW, body weight; CPI, crude protein intake; ME, metabolisable energy; TDN, total digestible nutrient; ADG, average daily gain; FCE, feed conversion efficiency.

1) Fat levels.

2) Protein levels.

A,B Capital letters in the same row, differ at p<0.05 by least square means for fat levels effect.

x,y Letters in the same row, differ at p<0.05 by least square means for protein levels effect.
